# Surface Molecules Involved in Avian T-Cell Progenitor Migration and Differentiation

**DOI:** 10.1155/2000/13850

**Published:** 2000

**Authors:** C. Ody, S. Alais, C. Corbel, K. M. McNagny, T. F. Davison, O. Vainio, B. A. Imhof, D. Dunon

**Affiliations:** 1 Department of Pathology Centre Médical Universitaire, 1 Rue Michel Servet Geneva 4 CH 1211 Switzerland; 2 CNRS UMR 7622 Adhesion et Migration Cellulaire Université Pierrre et Marie Curie 9 Quai St Bernard Paris Cedex 05 F 75272 France; 3 lnstitut d'Embryologie du CNRS 49bis av. De la Belle Gabrielle F 94736 Nogent-sur-Marne France; 4 Biomedical Research Center Vancouver Canada; 5 lnstitute for Animal Disease Research Houghton laboratory Huntingdon Cambs United Kingdom; 6 Department of Medical Microbiology University of Turku Kiinamyllynkatu 13 Turku Fin-20520 Finland; 7 Department of Pathology Centre Médical Universitaire, 1 Rue Michel Servet CH 1211 Geneva 4 Switzerland

**Keywords:** Embryogenesis, Hemopoiesis, T-cell progenitors, Surface molecules


*Developmental Immunology*, 2000, Vol. 7(2-4), pp. 267-277
Reprints available directly from the publisher
Photocopying permitted by license only

© 2000 OPA (Overseas Publishers Association)
N.V. Published by license under the
Harwood Academic Publishers imprint,
part of the Gordon and Breach Publishing Group.
Printed in Malaysia

### Surface Molecules Involved in Avian T-Cell Progenitor Migration and Differentiation

C. ODY[a*], S. ALAIS[b], C. CORBEL[c], K.M. McNAGNY[d], T.F. DAVISON[e], O. VAINIO[f], B.A. IMHOF[a†] and D. DUNON[b]

[a]*Department of Pathology, Centre Médical Universitaire, 1, Rue Michel Servet, CH 1211 Geneva 4, Switzerland,* [b]*CNRS UMR 7622 Adhesion et Migration Cellulaire, Université Pierrre et Marie Curie, 9 Quai St Bernard F 75272 Paris Cedex 05, France,* [c]*Institut d'Embryologie du CNRS, 49bis av. De la Belle Gabrielle, F 94736 Nogent-sur-Marne, France,* [d]*Biomedical Research Center, Vancouver, Canada,* [e]*Institute for Animal Disease Research, Houghton laboratory, Huntingdon, Cambs, England and* [f]*Department of Medical Microbiology, University of Turku, Kiinamyllynkatu 13, Fin-20520 Turku, Finland*

*Keywords:* Embryogenesis, Hemopoiesis, T-cell progenitors, Surface molecules

### ORIGIN AND MIGRATION OF T-CELL PROGENITORS DURING ONTOGENY

Comparative developmental studies are very informative with regard to the evolution of the immune system in vertebrates. The avian model offers several advantages for the study of T cell development: (i) T and B cells undergo differentiation in specialized central lymphoid organs, T cells in the thymus, and B cells in the bursa of Fabricius, (ii) a large number of precisely staged embryos can be easily obtained, (iii) the embryo is large enough for experimental manipulation, and (iv) the general scheme of Tcell ontogeny is similar in birds and mammals with the exception of the fetal liver which is not hemopoietic in birds. Studies performed in chick-quail chimeras show that the thymus of birds is colonized in three waves during embryogenesis and just after hatching. These waves start at day 6, day 12 and day 18 of embryonic development (E6, E12, E18) respectively (Coltey *et al.*, 1989; Coltey *et al.*, 1987; Jotereau and Le Douarin, 1982). The duration of these waves is of around 2 days and they are separated by periods refractory for thymus colonization. T-cell progenitors first originate from para-aortic mesoderm at the level of the ducts of Cuvier in E3 chicken embryos (Cormier and Dieterlen-Lievre, 1988; Dieterlen-Lievre *et al.*, 1996; Pardanaud *et al.*, 1996). During the second and third wave of thymus colonization, T cell progenitors are found in the bone marrow where they express various markers, some of which are adhesion molecules, including HEMCAM, BEN, CD44, thrombomucin and αIIbβ3 integrin.

The available evidence to date suggests that hemopoietic progenitors emerge in situ at three locations during chicken embryogenesis: the yolk sac, the aortic foci, and the allantois (Caprioli *et al.*, 1998; Cormier *et al.*, 1986; Dieterlen-Lievre and Martin, 1981; Moore and Owen, 1967). The other hemopoietic Anlagen that successively harbor progenitors during embryogenesis, such as the bone marrow, the spleen and the thymus may simply provide an environment

* Proofs should be sent to: Christiane Ody at Department of Pathology, Centre Médical Universitaire, 1, Rue Michel Servet, CH 1211 Geneva 4, Switzerland, Fax: ++41 22 702 57 46 E-mail: christiane.ody@medecine.unige.ch
† Correspondence should be addressed to: Beat A. Imhof at Department of Pathology, Centre Medical Universitaire, 1, Rue Michel Servet, CH 1211 Geneva 4, Switzerland.

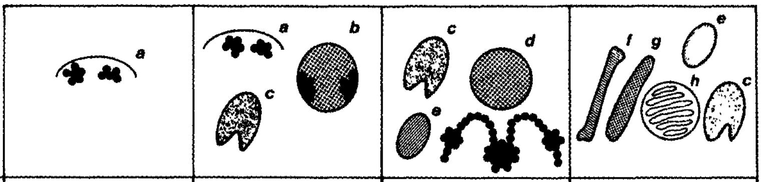

| | | | | | |
|---|---|---|---|---|---|
| chicken | day of development | 1 | 3-4 | 6-8 | 9-11 |
| | location | yolk sac[a] | yolk sac<br>basolateral intra-aorta[b] | dorsal mesentery, ventral to aorta[d]<br>thymus[e] | bone marrow[f]<br>spleen[g]<br>bursa of fabricius[h]<br>thymus |
| mouse | day of development | 8 | 10.5-11.5 | 12.5-13.5 | 14.5-16 |
| | location | yolk sac | fetal liver[c]<br>para-aortic splanchnopleura | fetal liver<br>thymus | bone marrow<br>spleen<br>fetal liver<br>thymus |

FIGURE 1 Sites of emergence of hemopoietic progenitor cells during embryogenesis. Comparison between mouse and chicken where lymphoid progenitors, presumably circulating in the blood-stream, settle and give rise to a differentiated progeny. The first T-cell progenitors that are transported close to the thymus leave the blood circulation through the jugular vein. They enter the non vascularized thymus Anlage through the capsule (Dunon *et al.*, 1993; Savagner *et al.*, 1986). After vascularization of the thymus, progenitors may then enter at the corticomedullary junction or between thymic lobules (Dunon *et al.*, 1997). When T-cell progenitors enter the perivascular space after invasive migration through the pericytic/epithelial basal membrane, they interact with the thymic microenvironment and undergo differentiation (Fig. 1). Based on a sensitive in vivo thymus reconstitution assay (see below), the number and frequency of T-cell progenitors in peripheral blood, para-aortic foci, bone marrow, and spleen have been quantified during ontogeny. The progenitors of the first wave colonize the embryonic thymus stem from the para-aortic foci and those of the second and third waves originate from bone marrow (Dunon *et al.*, 1999). During these latter waves, T cell progenitors are encountered in the bone marrow and spleen. However, the spleen, in contrast to the bone marrow, contains progenitors which are unable to home to the thymus via the blood stream. Each wave of thymus colonization correlates with the presence of a peak of progenitors in peripheral blood, whereas almost no progenitors are detected in the blood during the periods defined previously as refractory for thymus colonization (Fig. 2). Moreover, intravenous injection of T cell progenitors show that they are able to home into the thymus without delay even during the so-called refractory periods. These findings demonstrate that the blood delivery of T cell progenitors plays a major role in the thymus colonization kinetics during embryogenesis (Dunon *et al.*, 1999).

### IDENTIFICATION OF T-CELL PROGENITORS

Embryonic T-cell progenitors are identified by their ability to differentiate into T cells after intrathymic injection. In brief, blood cells or FACS sorted bone marrow cells are injected into thymi of irradiated congenic animals. The degree of chimerism of the host thymus is subsequently measured and correlated with the number of donor progenitors initially injected. This assay has been used to identify T cell progenitors expressing new cell surface molecules. Some of these molecules are

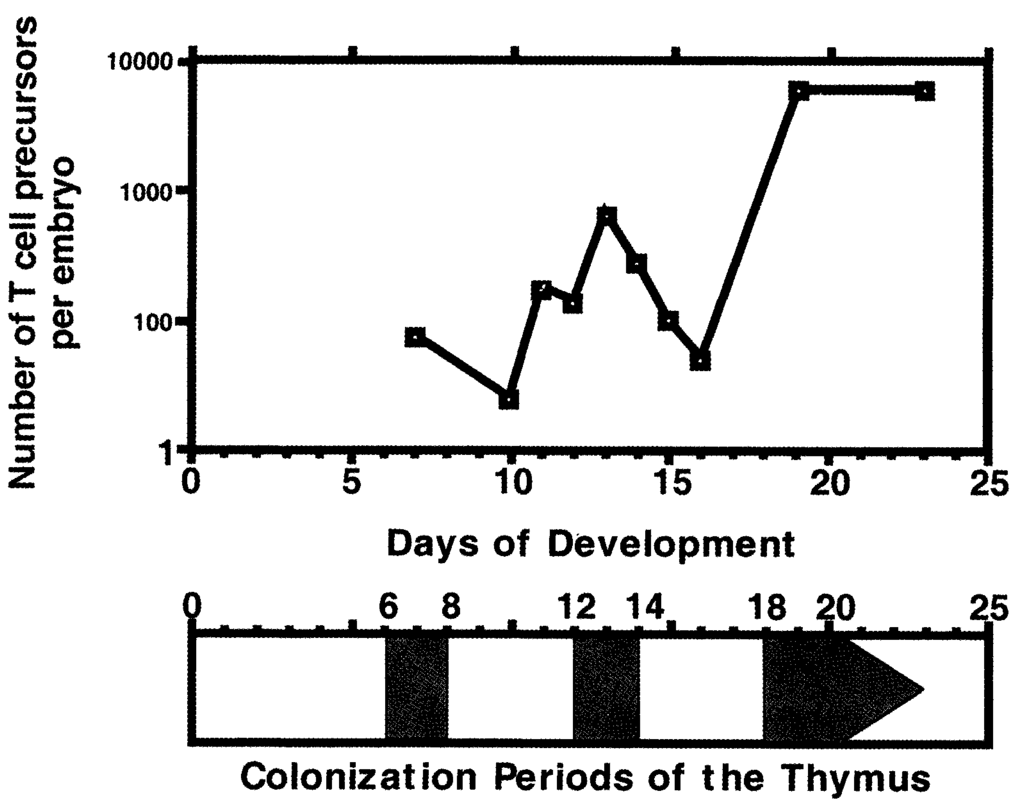

FIGURE 2 Quantification of T-cell progenitors in chicken embryonic blood involved in adhesion and/or signal transduction. In the chicken, they include c-kit, HEMCAM, BEN, αIIbβ3, ChT1, MHC class II, CD44, and thrombomucin (Fig. 3). The VEGFRII positive cells from the mesoderm of chicken embryos at the gastrulation stage, the so called hemangioblasts (Eichmann *et al.*, 1997) are not able to give rise to mature T cells in this system (C. Ody unpublished data), indicating the requirement for an additional maturation step, before they are able to differentiate in the thymic environment.

### T-cell Progenitors Surface Markers

### *C-kit*

The c-kit protein has five Ig like domains, linked to a transmembrane and a tyrosine kinase domain, and is closely related to the Platelet derived growth factor receptor. This 140 to 160 kDa protein becomes activated upon occupancy by its specific ligand, stem cell factor (SCF) or by antibody crosslinking. This tyrosine kinase receptor was among the first molecules to be described on hemopoietic cells in mammals, and transplantation experiments with c-kit positive bone marrow cells clearly demonstrate the presence of c-kit on primitive hemopoietic progenitors (Morrison *et al.*, 1997; Visser *et al.*, 1993). Recently, c-kit has also been found on pro-T cells in mammals (Di Santo and Rodewald, 1998). In the chicken, the less primitive T-cell progenitors, which are able to differentiate in the thymic environment, are also c-kit positive population (Katevuo *et al.*, 1999; Vainio *et al.*, 1996). The critical role of this receptor in hemopoiesis is well established following the identification of the genetic molecular structure

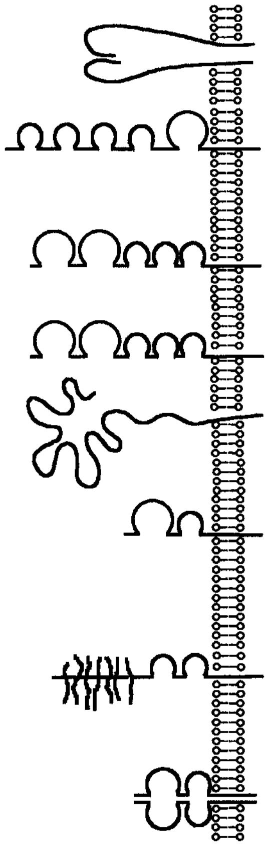

| MW kDa | Interaction with Endothelium | Interaction with ECM | Signaling | Molecule |
|---|---|---|---|---|
| 125/105 | n.d. | + | + | Integrin αIIbβ3 |
| 145 | + | - | + | c-kit |
| 98 | + | + | + | HEMCAM/ MCAM |
| 98 | n.d. | n.d. | n.d. | BEN |
| 80-95 | + | + | + | CD44 |
| 63 | unlikely | n.d. | + | ChT1 |
| 150 | n.d. | n.d. | n.d. | Thrombomucin |
| 34/29 | - | - | + | MHC class II |

FIGURE 3 Description of some molecules present on chicken T-cell progenitors. nd = not determined defect in the W and SI mouse strains: these mice have mutations in either the c-kit receptor or in its ligand (SCF), and they display a wide range of hemopoietic disorders not selectively affecting the T cell compartment (Chabot *et al.*, 1988; Geissler *et al.*, 1981; Huang *et al.*, 1990). So, c-kit mutations are not sufficient to suppress T cell development and it is necessary to cointroduce a mutation in the common cytokine receptor γ chain to fully abrogate T cell development. These mutations selectively affect the T cell compartment leaving the B cell compartment only mildly diminished (Rodewald *et al.*, 1997)). The γ chain is common to many interleukin receptors, but among these, only the IL-7 receptor seem important, since its knockout induces a reduction in thymic cellularity comparable to that observed in the γchain knock out mouse (Peschon *et al.*, 1994). This correlates with the presence of the IL-7 receptor on the common lymphoid progenitor cell in murine bone marrow (Kondo *et al.*, 1997).

### HEMCAM

HEMCAM (hemopoietic *c*ell *a*dhesion *m*olecule) is an adhesion molecule belonging to the immunoglobulin superfamily with a V-V-C2-C2-C2 Ig domain structure (Vainio *et al.*, 1996). HEMCAM positive bone marrow cells coexpressing c-kit can differentiate into T, myeloid and erythroid cells in vitro, suggesting that multipotent hemopoietic stem cells express this adhesion molecule. HEMCAM expression is not restricted to cells of the hemopoietic lineages, since this molecule is also expressed at high levels on endothelial cells in many tissues, on myocytes, and on the epithelial cells of the bursa of Fabricius. HEMCAM is identical to the chicken gicerin, a molecule involved in neurite outgrowth and Wilm's kidney tumor progression (Taira *et al.*, 1994; Takaha *et al.*, 1995). It is also homologous to MUC18/MCAM a human molecule involved in melanoma progression and metastasis (Johnson *et al.*, 1996; Lehmann *et al.*, 1989). There are three mRNA splice variants, one with a short cytoplasmic tail, another with a long tail and the third one lacking the transmembrane and cytoplasmic regions. The two transmembrane HEMCAM/gicerin isoforms are detected by immunoprecipitation and are differentially expressed in the developing nervous and immune systems. Initially, HEMCAM/gicerin was identified as a binding protein for the neurite outgrowth factor (NOF) a molecule of the laminin family (Hayashi and Miki, 1985; Taira *et al.*, 1994). In addition, HEMCAM promotes cell-cell adhesion probably through both heterophilic and homophilic binding. Several studies now suggest that HEMCAM might also transduce a signal (Anfosso *et al.*, 1998) which could regulate cell adhesion on laminin-1 (Alais et al., in preparation).

### BEN

BEN (*b*ursal *e*pithelium and *n*eurons) a surface glycoprotein also known as DM-GRASP and SC1 belongs to the same subfamily of adhesion molecules as HEMCAM, exhibiting a V-V-C2-C2-C2 Ig domain structure (Pourquie *et al.*, 1992). Its expression is tightly developmentally regulated in several cell types of the nervous and hemopoietic systems and in certain epithelia. BEN is expressed on hemopoietic cells as early as E7 and by E9 in the thymus (Corbel *et al.*, 1992). In the spleen BEN expression parallels the myelopoietic activity. During embryonic life and after hatching, 30–60% of thymocytes are BEN positive. In the embryo, most of the BEN positive thymocytes do not express CD3 and may be considered as undifferentiated T-cells. BEN is also present on bone marrow cells including the c-kit positive subpopulation, which contains T-cell progenitors and stem cells. In the E13 embryo, all the c-kit positive cells are also BEN positive (Fig. 4). In the adult chicken, the population of BEN-positive cells includes myeloid and erythroid progenitor cells. BEN expression is lost as progenitor cells proliferate and differentiate to develop into mature colonies in vitro. BEN is required for in vitro myeloid but not erythroid colony formation as shown by the effect of anti-BEN monoclonal antibody treatment (Corbel *et al.*, 1996). BEN interacts in a homophilic way and these interactions are not affected by its glycosylation status. In addition, Ng-CAM has been suggested as ligand for BEN (DeBernardo and Chang, 1996). ALCAM, the mammalian homologue of BEN, which is expressed on activated T lymphocytes, has been identified as a CD6 ligand (Bowen *et al.*, 1995). ALCAM-CD6 interactions are very likely involved in thymocyte-thymic epithelium interactions as well as in the binding of T and B cells to activated leukocytes. BEN might play a role in the migration of T-cell progenitors from the bone marrow to the thymus. As suggested by the in vitro inhibition studies, it may also be involved in the first step of T cell maturation possibly through interaction with the thymic epithelium.

### $\alpha IIb\beta 3$ Integrin

For a long time, the $\alpha IIb\beta 3$ integrin has been thought to be specific for the megakaryocytic lineage (Naik and Parise, 1997). Recently however, it was found that this integrin is also present on hemopoietic progenitors capable of differentiating into T cells and into cells of the myeloid lineages (Ody *et al.*, 1999).

During embryogenesis αIIbβ3 positive progenitors can be found as early as E3–4,5 in the para-aortic region. Later on in development and in the adult this integrin is coexpressed with c-kit on hemopoietic progenitors in the bone marrow. Expression is lost upon differentiation. In mice bearing a conditional αIIb knockout transgene, suppression of αIIbβ3 expression induces a severe reduction in the potential of bone marrow cells to generate mixed colonies in CFU assays and a marked thrombocytopenia (Tronik-Le Roux *et al.*, 1995). These results clearly indicate that this molecule also plays a pivotal role in the development of different hemopoietic lineages. The integrin αIIbβ3 binds to extracellular matrix molecules containing the minimal amino acid sequence RGD with a preference for fibrinogen as described with platelets (Naik and Parise, 1997). Similar to other integrins, the ligand binding depends on the previous activation of αIIbβ3 by an inside-out signal transduction pathway (Pelletier *et al.*, 1995). The exact role of αIIbβ3 in thymus homing and T-cell progenitors differentiation remains to be determined.

### ChT1

ChT1 is a transmembrane molecule well conserved through evolution (DuPasquier and Chretien, 1996), which belongs to the large ChT1 Ig supergene subfamily with one V- and one C2 extracellular domain (Katevuo *et al.*, 1999). JAM, CRAM-1 and CTX are related molecules (Aurrand-Lions, M. unpublished data). It is expressed by hemopoietic progenitors in the bone marrow during embryogenesis. It is present on 90% of thymocytes and in the blood on recent thymic emigrants. It is also expressed by splenic lymphocytes, which have recently rearranged their TCR genes as indicated by their content in DNA circles created by the αβ and γδ TCR gene rearrangements (Kong *et al.*, 1998). Treatment of thymic organ cultures with anti-ChT1-antibodies, blocked T cell differentiation at the level of the immature lymphocyte. Present data suggest that this molecule is involved in an early T cell differentiation step, preceding CD3, CD4 and CD8 expression (Katevuo *et al.*, 1999). The time-restricted expression on recent thymic emigrants is extremely useful allowing the selective study of these naive T cells at any stage of embryogenesis or in the adult (Kong *et al.*, 1998).

### MHC class II

In the c-kit positive population of the bone marrow, the T-cell progenitors are restricted to the cells coexpressing the MHC class II beta chain molecule at their surface (Ody, unpublished data, Fig 4). This population is present in the embryo as well as in the young adult, although at lower number in the latter. The fact that the c-kit / MHC class II double positive progenitors are in the Rho (Rhodamine 123) high fraction (Ody et al. in preparation) showed that they belong to the less primitive progenitors already engaged in the differentiation process. Indeed, Rho binds to mitochondrial membranes of metabolically active cells (Johnson *et al.*, 1980). Thus, Rho low cells are in a resting state. After selection with the standard markers for murine HSC (*h*emopoietic *s*tem *c*ell), the long term repopulating cells i.e. the most primitive HSC are found in the Rho low fraction of the bone marrow, whereas cells present in the Rho high fraction have a time restricted repopulating ability (Spangrude *et al.*, 1995). Accordingly, all the c-kit / MHC class II double positive cells are found in the Rho high fraction of the bone marrow. The expression of the MHC class II beta chain molecule is lost when the progenitors differentiate into CD4 CD8 double positive cells in the thymic environment ( Ody et al. in preparation). The role of this transmembrane protein in T cell migration and maturation is not yet elucidated. Nevertheless, in the MHC class II knockout mice (Gosgrove *et al.*, 1991), the disorganization of the $CD4^{+}$cells in the thymic architecture is an indication for a role of the MHC class II molecule in T cell differentiation and migration unrelated to T cell selection. Moreover in vivo and in vitro studies performed on dogs (Hong *et al.*, 1995b), show that anti-MHC class II induces failure of autologous bone marrow transplant after lethal irradiation treatment and prevents CFU-GM formation. This is accompanied by an increase in intracellular $Ca^{++}$ but no change in the tyrosine phosphorylation pattern is detected (Hong *et al.*,

1995a). These results also suggest a more general role of the MHC class II molecule in the regulation of hemopoiesis, which appears to be completely unrelated to its role as a histocompatibility barrier (Deeg and Huss, 1993).

### CD44

The CD44 proteoglycan is a widely expressed cell surface protein on leukocytes and endothelial cells (Borland *et al.*, 1998; Kincade *et al.*, 1997). CD44 mediates cell adhesion mainly by its binding to hyaluronic acid (HA), but it can also interact with chondroitin 4- sulphated serglycin, sulphated proteoglycans and the extracellular matrix molecules, collagen I and IV, laminin and fibronectin (Carter and Wayner, 1988; Jalkanen and Jalkanen, 1992; Peach *et al.*, 1993; Stamenkovic *et al.*, 1991). Mammalian CD44 isoforms are encoded by a single gene, containing 19 or 20 exons (Stamenkovic *et al.*, 1991). The enormous structural diversity of CD44 arises from the ability of cells to choose among a large number of mRNA splice options and from further glycosylation modifications. In the mouse, expression of CD44 by pro T cells in the bone marrow and the decrease in thymocytes number following injection of anti-CD44 antibodies suggest that CD44 plays a role in thymus homing (O'Neill, 1987; O'Neill, 1989; Spangrude and Scollay, 1990; Suniara *et al.*, 1999; Wu *et al.*, 1991). Thereby, the expression of CD44 by the thymic endothelium (Horst *et al.*, 1990) may also play a role. Moreover, CD44 is involved in progenitor interaction with the bone marrow stroma and in maturation of lymphoid progenitors. Accordingly, in the embryonic chicken bone marrow, CD44 is expressed by different cell populations at different levels. Most of the CD44 / c-kit double positive cells express CD44 at a high level. (Fig. 4). On mature T cells, CD44 seems to be involved in immune responses. It is the chondroitin 4-sulphated serglycin-CD44 interaction that provides a costimulatory signal to mouse cytotoxic lymphocytes (Lesley *et al.*, 1993; Miyake *et al.*, 1990). The chondroitin 4-sulphated serglycin-CD44 interaction may also be associated with MHC class II molecules. Such interactions could stimulate class II-dependent allogenic and mitogenic T cell responses (Naujokas *et al.*, 1993; Toyama-Sorimachi and Miyasaka, 1994). Interaction between CD44 and MHC class II might also play a role in the proliferation and/or differentiation of T cell progenitors since both molecules are present on these progenitors.

### Podocalyxin-like protein Thrombomucin

The Podocalyxin-like protein is a 140 kDa transmembrane sialomucin that was first identified as a marker of podocytes in the Kidney and vascular endothelia (Kershaw *et al.*, 1997; Kershaw *et al.*, 1995). The core protein has an estimated molecular weight of 55 kDa and contains putative sites for N- and O-glycosylation. Comparison of avian thrombomucin and mammalian Podocalyxin-like sequences shows a high degree of identity in the transmembrane and intracellular domains with a lower degree of identity in the extracellular domain (Kershaw *et al.*, 1997; McNagny *et al.*, 1997). Comparison with protein data base revealed structure and sequences similarities between thrombomucin and CD34 (Mc Nagny 1997; Sassetti 1998). The Podocalyxin-like protein is expressed at the basal side of podocytes in the glomeruli of the kidney as well as on some vascular endothelia (Kershaw *et al.*, 1997; Kershaw *et al.*, 1995). In addition, the avian thrombomucin is expressed on hematopoietic progenitors in the yolk sac and the bone marrow as well as the thrombocytes (McNagny *et al.*, 1997). In the embryonic bone marrow, there is a c-kit intermediate population, which is thrombomucin positive (Fig. 4). The T cell potential of this population has not yet been determined, but expression of thrombomucin on chicken lymphoid cells including T-cell progenitors has already been suggested (Lampisuo *et al.*, 1998; Lampisuo *et al.*, 1999). Podocalyxin-like protein is a ligand of L-selectin and the purified protein is able to support the tethering and rolling of lymphocytes under physiological flow conditions (Sassetti *et al.*, 1998). This makes it a good candidate for being a major player in the homing of T cell progenitors to the thymus during embryogenesis and early adult life.

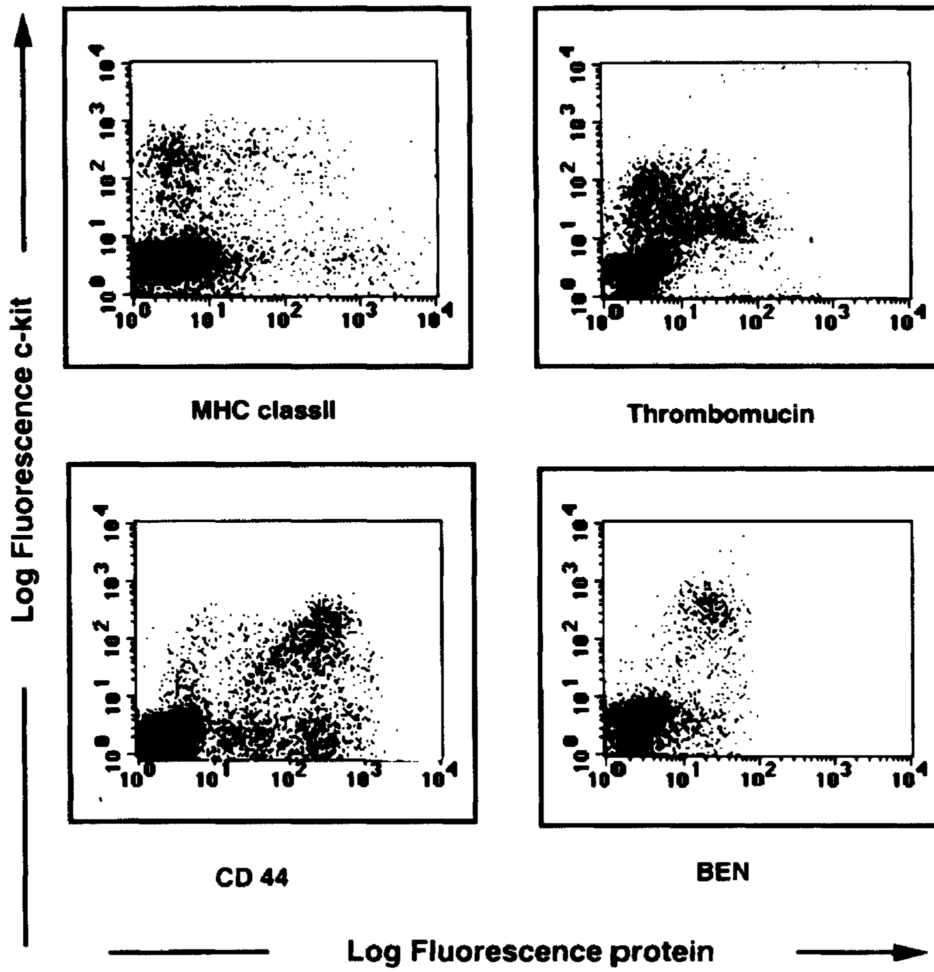

FIGURE 4 Flow cytometric analysis of double stained E13 chicken bone marrow cells

### CONCLUSIONS

The molecules described above have been detected on hemopoietic progenitors of birds and mammals. This denotes a high conservation through evolution, which could be linked to fundamental functions of these molecules. Though the immune system is very well conserved from birds to mammals, there appear to be additional and perhaps functionally less critical molecules, which are found exclusively in mammals. For instance, ChT1 was first cloned in xenopus (DuPasquier and Chretien, 1996), then independently in chicken (Katevuo *et al.*, 1999) and finally, related molecules have been identified in mouse and human (Aurrand-Lions M. submitted and in preparation). In contrast, PECAM, an adhesion molecule present on platelets, endothelial cells, most leukocytes (DeLisser *et al.*, 1993) and on hemopoietic progenitors (Ling *et al.*, 1997), has only been identified in higher mammals and has not been found so far in chicken in spite of many attempts. This finding is consistent with the apparent functional redundancy of PECAM-1 demonstrated by the absence of any major hemopoietic disorder in the PECAM knockout mice (Duncan *et al.*, 1999). On the other hand, the integrin αIIbβ3 and c-kit, which are highly conserved through evolution certainly play fundamental roles in the hemopoietic system. This is shown by the dramatic effects resulting from mutagenesis (Morrison-Graham and Takahashi, 1993), gene deletion (Tronik-Le Roux *et al.*, 1995) or antibody treatment (Berridge *et al.*, 1985). Thus avian system is very useful for the evaluation of unknown molecules as a bridge between organisms distant in the evolutionary tree. The avian model can also help in the understanding of the different mechanisms underlying hemopoiesis. For instance, the presence of HEMCAM on hemopoietic progenitors has been identified thanks to work performed on the chicken (Vainio *et al.*, 1996), whereas its earlier identification in human was related to melanoma progression (Lehmann *et al.*, 1989). Thus characterization of cell surface molecules expressed on T cell progenitors in birds and mammals are complementary and might help to improve our knowledge of the fundamental molecules involved in T cell migration, thymus homing and T cell differentiation.

### Acknowledgements

The authors wish to thank Caroline Johnson-Leger for critical reading of the manuscript.

This work was supported by

- the Swiss National Foundation, grant: 3100–049241.96
- the Human Frontier Science Program, grant: RG 0366 / 1996-M
- the Association pour la recherche contre le Cancer: ARC-9738

### References

Anfosso, F., Bardin, N., Frances, V., Vivier, E., Camoin-Jau, L., Sampol, J., and Dignat-George, F. (1998). Activation of human endothelial cells via S-endo-1 antigen (CD146) stimulates the tyrosine phosphorylation of focal adhesion kinase p125(FAK). *J Biol Chem* **273**, 26852–6.

Berridge, M. V., Ralph, S. J., and Tan, A. S. (1985). Cell-lineage antigens of the stem cell-megakaryocyte-platelet lineage are associated with the platelet IIb-IIIa glycoprotein complex. *Blood* **66**, 76–85.

Borland, G., Ross, J. A., and Guy, K. (1998). Forms and functions of CD44. *Immunology* **93**, 139–48.

Bowen, M. A., Patel, D. D., Li, X., Modrell, B., Malacko, A. R., Wang, W. C., Marquardt, H., Neubauer, M., Pesando, J. M., Francke, U., and et al. (1995). Cloning, mapping, and characterization of activated leukocyte-cell adhesion molecule (ALCAM), a CD6 ligand. *J Exp Med* **181**, 2213–20.

Caprioli, A., Jaffredo, T., Gautier, R., Dubourg, C., and Dieterlen-Lievre, F. (1998). Blood-borne seeding by hematopoietic and endothelial precursors from the allantois. *Proc Natl Acad Sci U S A* **95**, 1641–6.

Carter, W. G., and Wayner, E. A. (1988). Characterization of the class III collagen receptor, a phosphorylated, transmembrane glycoprotein expressed in nucleated human cells. *J Biol Chem* **263**, 4193–201.

Chabot, B., Stephenson, D. A., Chapman, V. M., Besmer, P., and Bernstein, A. (1988). The proto-oncogene c-kit encoding a transmembrane tyrosine kinase receptor maps to the mouse W locus. *Nature* **335**, 88–9.

Coltey, M., Bucy, R. P., Chen, C. H., Cihak, J., Losch, U., Char, D., Le Douarin, N. M., and Cooper, M. D. (1989). Analysis of the first two waves of thymus homing stem cells and their T cell progeny in chick-quail chimeras. *J Exp Med* **170**, 543–57.

Coltey, M., Jotereau, F. V., and Le Douarin, N. M. (1987). Evidence for a cyclic renewal of lymphocyte precursor cells in the embryonic chick thymus. *Cell Differ* **22**, 71–82.

Corbel, C., Cormier, F., Pourquie, O., and Bluestein, H. G. (1992). BEN, a novel surface molecule of the immunoglobulin superfamily on avian hemopoietic progenitor cells shared with neural cells. *Exp Cell Res* **203**, 91–9.

Corbel, C., Pourquie, O., Cormier, F., Vaigot, P., and Le Douarin, N. M. (1996). BEN/SC1/DM-GRASP, a homophilic adhesion molecule, is required for in vitro myeloid colony formation by avian hemopoietic progenitors. *Proc Natl Acad Sci U S A* **93**, 2844–9.

Cormier, F., de Paz, P., and Dieterlen-Lievre, F. (1986). In vitro detection of cells with monocytic potentiality in the wall of the chick embryo aorta. *Dev Biol* **118**, 167–75.

Cormier, F., and Dieterlen-Lievre, F. (1988). The wall of the chick embryo aorta harbours M-CFC, G-CFC, GM-CFC and BFU-E. *Development* **102**, 279–85.

DeBernardo, A. P., and Chang, S. (1996). Heterophilic interactions of DM-GRASP: GRASP-NgCAM interactions involved in neurite extension. *J Cell Biol* **133**, 657–66.

Deeg, H. J., and Huss, R. (1993). Major histocompatibility complex class II molecules, hemopoiesis and the marrow microenvironment. *Bone Marrow Transplant* **12**, 425–30.

DeLisser, H. M., Yan, H. C., Newman, P. J., Muller, W. A., Buck, C. A., and Albelda, S. M. (1993). Platelet/endothelial cell adhesion molecule-1 (CD31)-mediated cellular aggregation involves cell surface glycosaminoglycans. *J Biol Chem* **268**, 16037–46.

Di Santo, J. P., and Rodewald, H. R. (1998). In vivo roles of receptor tyrosine kinases and cytokine receptors in early thymocyte development. *Curr Opin Immunol* **10**, 196–207.

Dieterlen-Lievre, F., Godin, I., and Pardanaud, L. (1996). Ontogeny of hematopoiesis in the avian embryo: a general paradigm. *Curr Top Microbiol Immunol* **212**, 119–28.

Dieterlen-Lievre, F., and Martin, C. (1981). Diffuse intraembryonic hemopoiesis in normal and chimeric avian development. *Dev Biol* **88**, 180–91.

Duncan, G. S., Andrew, D. P., Takimoto, H., Kaufman, S. A., Yoshida, H., Spellberg, J., Luis de la Pompa, J., Elia, A., Wakeham, A., Karan-Tamir, B., Muller, W. A., Senaldi, G., Zukowski, M.

M., and Mak, T. W. (1999). Genetic evidence for functional redundancy of Platelet/Endothelial cell adhesion molecule-1 (PECAM-1): CD31-deficient mice reveal PECAM-1- dependent and PECAM-1-independent functions. *J Immunol* **162**, 3022–30.

Dunon, D., Allioli, N., Vainio, O., Ody, C., and Imhof, B. A. (1999). Quantification of T-cell progenitors during ontogeny: thymus colonization depends on blood delivery of progenitors. *Blood* **93**, 2234–43.

Dunon, D., Courtois, D., Vainio, O., Six, A., Chen, C. H., Cooper, M. D., Dangy, J. P., and Imhof, B. A. (1997). Ontogeny of the immune system: gamma/delta and alpha/beta T cells migrate from thymus to the periphery in alternating waves. *J Exp Med* **186**, 977–88.

Dunon, D., Ruiz, P., and Imhof, B. A. (1993). Pro-T cell homing to the thymus. *Curr Top Microbiol Immunol* **184**, 139–50.

DuPasquier, L., and Chretien, I. (1996). CTX, a new lymphocyte receptor in Xenopus, and the early evolution of Ig domains. *Res Immunol* **147**, 218–26.

Eichmann, A., Corbel, C., Nataf, V., Vaigot, P., Breant, C., Le Douarin, N. M. (1997). Ligand-dependent development of the endothelial and hemopoietic lineages from embryonic mesodermal cells expressing vascular endothelial growth factor receptor 2. *Proc Natl Acad Sci USA* **94**, 5141-5146.

Geissler, E. N., McFarland, E. C., and Russell, E. S. (1981). Analysis of pleiotropism at the dominant white-spotting (W) locus of the house mouse: a description of ten new W alleles. *Genetics* **97**, 337–61.

Gosgrove, D., Gray, D., Dierich, A., Kaufman, J., Lemeur, M., Benoist, C., and Mathis, D. (1991). Mice lacking MHC class II molecules. *Cell* **66**, 1051–66.

Hayashi, Y., and Miki, N. (1985). Purification and characterization of a neurite outgrowth factor from chicken gizzard smooth muscle. *J Biol Chem* **260**, 14269–78.

Hong, D. S., Beckham, C., Huss, R., Lee, J. W., Hockenbery, D., Ledbetter, J. A., and Deeg, H. J. (1995a). Major histocompatibility complex class II-mediated inhibition of hematopoiesis in long-term marrow cultures involves apoptosis and is prevented by c-kit ligand [see comments]. *Blood* **86**, 3341–52.

Hong, D. S., Huss, R., Beckham, C., Hoy, C. A., Storb, R., and Deeg, H. J. (1995b). Major histocompatibility complex class II-mediated inhibition of hemopoiesis in vitro and in vivo is abrogated by c-kit ligand. *Transplant Proc* **27**, 642–3.

Horst, E., Meijer, C. J., Duijvestijn, A. M., Hartwig, N., Van der Harten, H. J., and Pals, S. T. (1990). The ontogeny of human lymphocyte recirculation: high endothelial cell antigen (HECA-452) and CD44 homing receptor expression in the development of the immune system. *Eur J Immunol* **20**, 1483–9.

Huang, E., Nocka, K., Beier, D. R., Chu, T. Y., Buck, J., Lahm, H. W., Wellner, D., Leder, P., and Besmer, P. (1990). The hematopoietic growth factor KL is encoded by the SI locus and is the ligand of the c-kit receptor, the gene product of the W locus. *Cell* **63**, 225–33.

Jalkanen, S., and Jalkanen, M. (1992). Lymphocyte CD44 binds the COOH-terminal heparin-binding domain of fibronectin. *J Cell Biol* **116**, 817–25.

Johnson, J. P., Rummel, M. M., Rothbacher, U., and Sers, C. (1996). MUC18: A cell adhesion molecule with a potential role in tumor growth and tumor cell dissemination. *Curr Top Microbiol Immunol* **213**, 95–105.

Johnson, L. V., Walsh, M. L., and Chen, L.B. (1980). Localization of mitochondria in living cells with rhodamine 123. *Proc Natl Acad Sci U S A* **77**, 990–4.

Jotereau, F. V., and Le Douarin, N. M. (1982). Demonstration of a cyclic renewal of the lymphocyte precursor cells in the quail thymus during embryonic and perinatal life. *J Immunol* **129**, 1869–77.

Katevuo, K., Imhof, B. A., Boyd, R., Chidgey, A., Bean, A., Dunon, D., Gobel, T. W., and Vainio, O. (1999). ChT1, an Ig superfamily molecule required for T cell differentiation. *J Immunol* **162**, 5685–94.

Kershaw, D. B., Beck, S. G., Wharram, B. L., Wiggins, J. E., Goyal, M., Thomas, P. E., and Wiggins, R. C. (1997). Molecular cloning and characterization of human podocalyxin-like protein. Orthologous relationship to rabbit PCLP1 and rat podocalyxin. *J Biol Chem* **272**, 15708–14.

Kershaw, D. B., Thomas, P. E., Wharram, B. L., Goyal, M., Wiggins, J. E., Whiteside, C. I., and Wiggins, R. C. (1995). Molecular cloning, expression, and characterization of podocalyxin-like protein 1 from rabbit as a transmembrane protein of glomerular podocytes and vascular endothelium. *J Biol Chem* **270**, 29439–46.

Kincade, P. W., Zheng, Z., Katoh, S., and Hanson, L. (1997). The importance of cellular environment to function of the CD44 matrix receptor. *Curr Opin Cell Biol* **9**, 635–42.

Kondo, M., Weissman, I. L., and Akashi, K. (1997). Identification of clonogenic common lymphoid progenitors in mouse bone marrow. *Cell* **91**, 661–72.

Kong, F., Chen, C. H., and Cooper, M. D. (1998). Thymic function can be accurately monitored by the level of recent T cell emigrants in the circulation. *Immunity* **8**, 97–104.

Lampisuo, M., Katevuo, K., and Lassila, O. (1998). Antigenic phenotype of early intra-embryonic lymphoid progenitors in the chicken. *Scand J Immunol* **48**, 52–8.

Lampisuo, M., Liippo, J., Vainio, O., McNagny, K. M., Kulmala, J., and Lassila, O. (1999). Characterization of prethymic progenitors within the chicken embryo. *Int Immunol* **11**, 63–9.

Lehmann, J. M., Riethmuller, G., and Johnson, J. P. (1989). MUC18, a marker of tumor progression in human melanoma, shows sequence similarity to the neural cell adhesion molecules of the immunoglobulin superfamily. *Proc Natl Acad Sci U S A* **86**, 9891–5.

Lesley, J., Hyman, R., and Kincade, P. W. (1993). CD44 and its interaction with extracellular matrix. *Adv Immunol* **54**, 271–335.

Ling, V., Luxenberg, D., Wang, J., Nickbarg, E., Leenen, P. J., Neben, S., and Kobayashi, M. (1997). Structural identification of the hematopoietic progenitor antigen ER- MP12 as the vascular endothelial adhesion molecule PECAM-1 (CD31). *Eur J Immunol* **27**, 509–14.

McNagny, K. M., Pettersson, I., Rossi, F., Flamme, I., Shevchenko, A., Mann, M., and Graf, T. (1997). Thrombomucin, a novel cell surface protein that defines thrombocytes and multipotent hematopoietic progenitors. *J Cell Biol* **138**, 1395–407.

Miyake, K., Underhill, C. B., Lesley, J., and Kincade, P. W. (1990). Hyaluronate can function as a cell adhesion molecule and CD44 participates in hyaluronate recognition. *J Exp Med* **172**, 69–75.

Moore, M. A., and Owen, J. J. (1967). Chromosome marker studies in the irradiated chick embryo. *Nature* **215**, 1081–2.

Morrison, S. J., Wandycz, A. M., Hemmati, H. D., Wright, D. E., and Weissman, I. L. (1997). Identification of a lineage of multipotent hematopoietic progenitors. *Development* **124**, 1929–39.

Morrison-Graham, K., and Takahashi, Y. (1993). Steel factor and c-kit receptor: from mutants to a growth factor system. *Bioessays* **15**, 77–83.

Naik, U. P., and Parise, L. V. (1997). Structure and function of platelet alpha IIb beta 3. *Curr Opin Hematol* **4**, 317–22.

Naujokas, M. F., Morin, M., Anderson, M. S., Peterson, M., and Miller, J. (1993). The chondroitin sulfate form of invariant chain can enhance stimulation of T cell responses through interaction with CD44. *Cell* **74**, 257–68.

Ody, C., Vaigot, P., Quere, P., Imhof, B. A., and Corbel, C. (1999). Glycoprotein IIb-IIIa is expressed on avian multilineage hematopoietic progenitor cells. *Blood* **93**, 2898–906.

O'Neill, H. C. (1987). Isolation of a thymus-homing Lyt-2-, L3T4-T-cell line from mouse spleen. *Cell Immunol* **109**, 222–30.

O'Neill, H. C. (1989). Antibody which defines a subset of bone marrow cells that can migrate to thymus. *Immunology* **68**, 59–65.

Pardanaud, L., Luton, D., Prigent, M., Bourcheix, L. M., Catala, M., and Dieterlen-Lievre, F. (1996). Two distinct endothelial lineages in ontogeny, one of them related to hemopoiesis. *Development* **122**, 1363–71.

Peach, R. J., Hollenbaugh, D., Stamenkovic, I., and Aruffo, A. (1993). Identification of hyaluronic acid binding sites in the extracellular domain of CD44. *J Cell Biol* **122**, 257–64.

Pelletier, A. J., Kunicki, T., Ruggeri, Z. M., and Quaranta, V. (1995). The activation state of the integrin alpha IIb beta 3 affects outside- in signals leading to cell spreading and focal adhesion kinase phosphorylation. *J Biol Chem* **270**, 18133–40.

Peschon, J. J., Morrissey, P. J., Grabstein, K. H., Ramsdell, F. J., Maraskovsky, E., Gliniak, B. C., Park, L. S., Ziegler, S. F., Williams, D. E., Ware, C. B., and et al. (1994). Early lymphocyte expansion is severely impaired in interleukin 7 receptor-deficient mice. *J Exp Med* **180**, 1955–60.

Pourquie, O., Corbel, C., Le Caer, J. P., Rossier, J., and Le Douarin, N. M. (1992). BEN, a surface glycoprotein of the immunoglobulin superfamily, is expressed in a variety of developing systems. *Proc Natl Acad Sci U S A* **89**, 5261–5.

Rodewald, H. R., Ogawa, M., Haller, C., Waskow, C., and DiSanto, J. P. (1997). Pro-thymocyte expansion by c-kit and the common cytokine receptor gamma chain is essential for repertoire formation. *Immunity* **6**, 265–72.

Sassetti, C., Tangemann, K., Singer, M. S., Kershaw, D. B., and Rosen, S. D. (1998). Identification of podocalyxin-like protein as a high endothelial venule ligand for L-selectin: parallels to CD34. *J Exp Med* **187**, 1965–75.

Savagner, P., Imhof, B. A., Yamada, K. M., and Thiery, J. P. (1986). Homing of hemopoietic precursor cells to the embryonic thymus: characterization of an invasive mechanism induced by chemotactic peptides. *J Cell Biol* **103**, 2715–27.

Spangrude, G. J., Brooks, D. M., and Tumas, D. B. (1995). Long-term repopulation of irradiated mice with limiting numbers of purified hematopoietic stem cells: in vivo expansion of stem cell phenotype but not function. *Blood* **85**, 1006–16.

Spangrude, G. J., and Scollay, R. (1990). Differentiation of hematopoietic stem cells in irradiated mouse thymic lobes. Kinetics and phenotype of progeny. *J Immunol* **145**, 3661–8.

Stamenkovic, I., Aruffo, A., Amiot, M., and Seed, B. (1991). The hematopoietic and epithelial forms of CD44 are distinct polypeptides with different adhesion potentials for hyaluronate-bearing cells. *Embo J* **10**, 343–8.

Suniara, R. K., Jenkinson, E. J., and Owen, J. J. (1999). Studies on the phenotype of migrant thymic stem cells. *Eur J Immunol* **29**, 75–80.

Taira, E., Takaha, N., Taniura, H., Kim, C. H., and Miki, N. (1994). Molecular cloning and functional expression of gicerin, a novel cell adhesion molecule that binds to neurite outgrowth factor. *Neuron* **12**, 861–72.

Takaha, N., Taira, E., Taniura, H., Nagino, T., Tsukamoto, Y., Matsumoto, T., Kotani, T., Sakuma, S., and Miki, N. (1995). Expression of gicerin in development, oncogenesis and regeneration of the chick kidney. *Differentiation* **58**, 313–20.

Toyama-Sorimachi, N., and Miyasaka, M. (1994). A novel ligand for CD44 is sulfated proteoglycan. *Int Immunol* **6**, 655–60.

Tronik-Le Roux, D., Roullot, V., Schweitzer, A., Berthier, R., and Marguerie, G. (1995). Suppression of erythro-megakaryocytopoiesis and the induction of reversible thrombocytopenia in mice transgenic for the thymidine kinase gene targeted by the platelet glycoprotein alpha IIb promoter [published erratum appears in J Exp Med 1995 Oct 1;182(4):1177]. *J Exp Med* **181**, 2141–51.

Vainio, O., Dunon, D., Aissi, F., Dangy, J. P., McNagny, K. M., and Imhof, B. A. (1996). HEMCAM, an adhesion molecule expressed by c-kit+ hemopoietic progenitors. *J Cell Biol* **135**, 1655–68.

Visser, J. W., Rozemuller, H., de Jong, M. O., and Belyavsky, A. (1993). The expression of cytokine receptors by purified hemopoietic stem cells. *Stem Cells (Dayt)* **11 Suppl 2**, 49–55.

Wu, L., Antica, M., Johnson, G. R., Scollay, R., and Shortman, K. (1991). Developmental potential of the earliest precursor cells from the adult mouse thymus. *J Exp Med* **174**, 1617–27.

